# Dasatinib inhibits primary melanoma cell proliferation through morphology-dependent disruption of Src-ERK signaling

**DOI:** 10.3892/ol.2012.1066

**Published:** 2012-12-10

**Authors:** JIANGHONG WU, XIN LIAO, BO YU, BING SU

**Affiliations:** 1 Department of Gastric Cancer and Soft Tissue Sarcoma Surgery, Fudan University Shanghai Cancer Center, Shanghai 200032;; 2 Department of Oncology, Shanghai Medical College, Fudan University, Shanghai 200032;; 3 Biomedical Research Institute, Shenzhen-PKU-HKUST Medical Center, Futian, Shenzhen, Guangdong 518036, P.R. China;; 4 Department of Dermatology, Peking University Shenzhen Hospital, Futian, Shenzhen, Guangdong 518036, P.R. China;; 5 Shenzhen Key Laboratory for Translational Medicine of Dermatology, Shenzhen-PKU-HKUST Medical Center, Futian, Shenzhen, Guangdong 518036, P.R. China;; 6 Shenzhen Key Discipline of Dermatology, Peking University Shenzhen Hospital, Futian, Shenzhen, Guangdong 518036, P.R. China

**Keywords:** melanoma, Src, ERK, dasatinib, U0126

## Abstract

New strategies for the treatment of advanced melanoma are urgently required. The RAS/RAF/MAPK pathway and c-Src are deregulated in the majority of malignant melanomas, suggesting that they may interact functionally and are involved in the development and progression of the malignancy. Preclinical studies have demonstrated variable inhibition of melanoma cell growth by dasatinib *in vitro*. Src may act through different downstream signaling pathways. In the present study, we demonstrate that dasatinib induces changes in cell morphology, characterized by an arborized and contracted appearance, and accompanied by a reduction in cell proliferation in primary melanoma cells. This morphological change is demonstrated to be associated with the inhibition of nuclear translocation of activated ERK1/2. Together, these results indicate that Src may promote cell proliferation through the activation of the ERK signaling pathway in melanoma oncogenesis.

## Introduction

The incidence of malignant melanoma has increased markedly over the past three decades, more rapidly than any other solid malignancy. Standard of care chemotherapeutic agents, such as dacarbazine and temozolomide, yield poor response rates of <20% (1). Therefore, new strategies for the treatment of advanced melanoma are urgently required.

Src tyrosine kinase family (SFK) members are known to be overexpressed and/or activated in many primary types of human cancer, typically through the mutational activation of upstream growth factor receptor tyrosine kinases (2). Increased protein levels and kinase activities of SFK have also been observed in melanoma (3,4). Dasatinib is a small molecule tyrosine kinase inhibitor that was initially isolated as a dual Src/ABL inhibitor (5), which has been approved by the Food and Drug Administration (FDA) for imatinib-resistant chronic myelogenous leukemia (CML) and Philadelphia chromosome-positive (Ph^+^) acute lymphoblastic leukemia (ALL) treatment (6,7). An abundance of studies support the anti-tumor effects of dasatinib in cancer prevention and treatment, including those concerned with triple-negative breast (8–10), gastric (11), pancreatic (12), head and neck and lung cancer cell lines (13), as well as with myeloid leukemia (14).

However, preclinical studies have demonstrated variable inhibition of melanoma cell growth by dasatinib *in vitro*. Eustace *et al* identified an IC_50_ value in the nanomolar range in only one out of five cell lines (15), Homsi *et al* demonstrated variable sensitivity in three cell lines (4), Buettner *et al* revealed little to no effect on viability (16) and Kluger *et al* demonstrated that two out of eight melanoma cell lines used in the study were growth inhibited by concentrations <300 nM, whereas the other six were significantly more resistant (17). Src may act through different downstream signaling pathways. Hence, the underlying regulatory mechanisms for the discrepancies in the antiproliferative effects require investigation.

The RAS/RAF/MAPK pathway is deregulated in >90% of malignant melanomas. MAPK activation is crucial for the development of melanocytic neoplasia, and a constitutive activation of this pathway has been associated with numerous types of cancer (18,19). Notably, Maat *et al* demonstrated a reduction in ERK1/2 activation in metastatic cell lines compared with that of primary uveal melanoma (UM) cell lines, and Src kinase was involved in the ERK1/2 activation (20). This suggests that Src may be involved by regulating the ERK signaling pathway in melanoma oncogenesis.

In the present study, we demonstrate that dasatinib induces changes in cell morphology, characterized by an arborized and contracted appearance, and accompanied by a reduction in cell proliferation in primary melanoma cells. This morphological change is associated with the restriction of ERK1/2 activity in the cytoplasmic compartment.

## Materials and methods

### Antibodies and reagents

The following primary antibodies (Ab) were used: Rabbit polyclonal antibody specific for GAPDH (Santa Cruz Biotechnology, Inc., Santa Cruz, CA, USA); Src, phospho-Src^Tyr416^, phospho-ERK1/2^Thr202/Tyr204^ and ERK1/2 (Cell Signaling Technology, Inc., Beverly, MA, USA). Dasatinib was a gift from Dr Irwin Gelman (Roswell Park Cancer Institute, Buffalo, NY, USA). The MEK1/2 inhibitor (U0126) was purchased from Calbiochem (San Diego, CA, USA).

### Cell culture

Melanoma cells were derived from primary melanoma known as Mel-p. The metastatic melanoma cell line A375 was obtained from the Typical Cell Culture Collection Committee of the Chinese Academy of Sciences. Cells were maintained in Dulbecco’s modified Eagle’s media (DMEM) supplemented with 10% fetal bovine serum (FBS).

### MTT assay

Cells (1,000 cells/well; 96-well plate) were incubated overnight at 37°C in 5% CO_2_, in media with 10% FBS. The following day, cells were treated with either a vehicle control (dimethylsulfoxide, DMSO) or varying concentrations of dasatinib/U0126, and allowed to grow for an additional 72 h. After 72 h, cell numbers were assessed by an MTT assay; 20 *μ*l of 5 mg/ml MTT was added to each well. Subsequently, the plate was incubated at 37°C and 5% CO_2_ for 4–5 h. The medium was then removed and 150 *μ*l of DMSO was added. The plate was then incubated in the same conditions as previously for 5 min. Proliferation was quantified by a plate reader at optical density (OD) of 570 nm. The cell growth inhibition was calculated as (T–T0)/(C–T0) × 100 (T, OD of the test well on exposure to the test drug; C, OD of the vehicle control well; T0, OD at time zero). The cell growth inhibition curve was generated by plotting cell growth inhibition against drug concentration, and IG_50_ was determined using GraphPad Prism 5 software (GraphPad Software, Inc., La Jolla, CA, USA).

### Cell morphology

Mel-p and A375 cells were plated overnight in 6-well dishes in the presence or absence of dasatinib (30 nM) or U0126 (10 *μ*M). The plates were photographed digitally using a phase-contrast microscope.

### Immunofluorescence analysis

Melanoma cells were plated on glass coverslips and treated with DMSO or 30 nM dasatinib for 24 h, and then washed twice with PBS. The cells were then fixed with 60% acetone/3.7% formaldehyde at −20°C for 20 min, and blocked with 3% bovine serum albumin (BSA) in PBS for 30 min at room temperature. Actin filaments were stained with rhodamine-labeled phalloidin (1:500; Sigma, St. Louis, MO, USA) and nuclei were stained with DAPI (1:500; Invitrogen Life Technologies; Carlsbad, CA, USA) for 1 h. Fluorescent images were captured using an Olympus inverted microscope equipped with a Roper CoolSnap HQ CCD camera (Metronet Technology Ltd. (Guangzhou, China). For p-ERK1/2 staining, melanoma cells were plated on glass coverslips and treated with DMSO or 30 nM dasatinib for 24 h, and serum-starved overnight by incubation with serum-free DMEM. The cells were stimulated with 10% FBS in DMEM at the times indicated in the specific figure legends and were immediately fixed with 60% acetone/3.7% formaldehyde at −20°C, following the procedure described previously.

### Western blot analysis

Cells grown in the presence or absence of dasatinib or U0126 at the indicated concentration were plated in 10-cm dishes and incubated with regular DMEM overnight, then lysed in RIPA buffer. Proteins (40 *μ*g per sample) were separated by SDS-PAGE, blotted onto PVDF membranes that were blocked for 1 h with 5% BSA in 1X Tris-buffered saline with 0.1% Tween-20 (TBST) and then probed as described. Digital imaging and signal quantification were performed using the Chemi-Genius2 Bio-Imager (Syngene, Frederick, MD, USA) using GeneTools software.

## Results and Discussion

### Dasatinib differentially inhibits cell growth in melanoma cell lines

Previous studies have demonstrated variable sensitivity to dasatinib in different melanoma cells. Recently, Maat *et al*(20) demonstrated that inhibition of Src led to the growth reduction of primary uveal melanoma cultures and cell lines, whereas metastatic cell line growth was only slightly reduced. It was suggested that Src may be involved in the initiation of melanoma oncogenesis. To test this hypothesis, two melanoma cell lines (Mel-p, primary melanoma cells and A375, metastatic melanoma cells) were examined for their sensitivity to dasatinib *in vitro* using an MTT assay. The IC_50_ values were calculated, following treatment with dasatinib for 72 h. Mel-p cells demonstrated robust growth inhibition with an IC_50_ value of 18.02 nM. Consistent with a previous study (4), A375 cells were less responsive with an IC_50_ of 762.4 nM. These results demonstrate that the inhibition of Src by dasatinib leads to the growth inhibition of primary melanoma cells.

### Dasatinib induces cell differentiation and remodels the actin cytoskeleton in Mel-p cells

**Figure 1.**
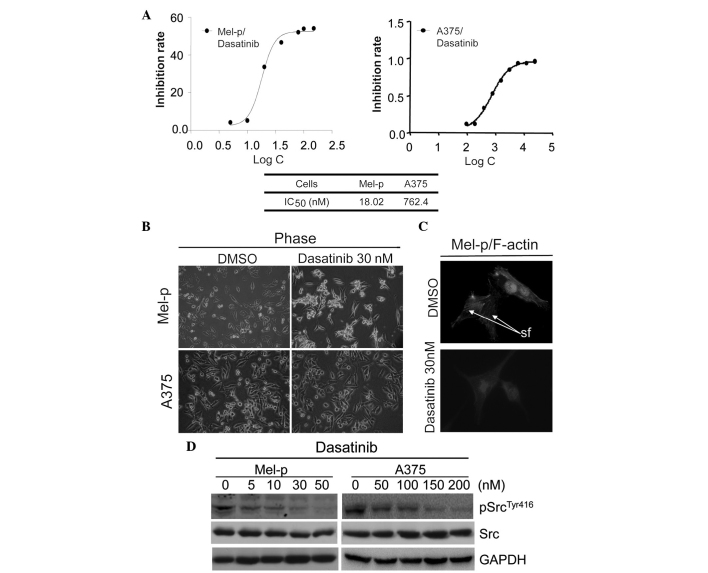
Dasatinib induces cell differentiation and remodels the actin cytoskeleton in Mel-p cells. (A) Mel-p and A375 cells were treated with various concentrations of dasatinib for 72 h. Cell viability was measured using the MTT assay. The IC_50_ values of dasatinib were determined. Results shown are representative of three independent experiments. (B) Morphological changes in Mel-p cells induced by dasatinib. Monolayer Mel-p (upper panel) and A375 (lower panel) cells were treated with 50 nM dasatinib or dimethylsulfoxide (DMSO) vehicle control for 24 h, then imaged by phase-contrast microscopy. (C) Mel-p cells were grown in the presence or absence of dasatinib, fixed and stained for F-actin (rhodamine-phalloidin) stress fibers (sf). (D) Dasatinib inhibited Src activation in both types of cells. Mel-p and A375 cells were treated with different doses of dasatinib. Whole cell lysates were immunoblotted for phospho-Src^Tyr416^ and total Src. GAPDH served as a loading control.

Notably, we observed that dasatinib treatment induced changes in the morphology of Mel-p cells, which normally present as flattened and extended cells. Upon dasatinib treatment at a concentration of 30 nM, the cells displayed a markedly different morphology that was characterized by an arborized and contracted appearance (Fig. 1B), which is recognized as a morphological indication of melanoma cell differentiation (21). The percentage of arborized cells following treatment with dasatinib (30 nM) overnight was counted. The results revealed that 70.2% of dasatinib-treated Mel-p cells were arborized in comparison to the control cells (2%). By contrast, no morphological changes were observed in the A375 cells treated with 30 nM of dasatinib (Fig. 1B), while only minor morphological changes were observed in the A375 cells treated with a higher concentration of dasatinib (≥200 nM) that clearly inhibited Src activation (Fig. 1D). These results suggest that Src differentially regulates melanoma cell morphology.

We further studied whether the remodeling of cytoskeletal components, such as microfilaments, was involved in the formation of dendrites in Mel-p cells. As demonstrated in Fig. 1C, in untreated Mel-p cells, actin was organized in stress fibers crossing the cytoplasm. Following treatment with 30 nM dasatinib for 24 h, the actin cytoskeletal structure was disrupted, creating a dense and compact cell body. This suggests that inhibition of cell proliferation by dasatinib is associated with changes in cell shape. Certain fundamental cellular processes (cell growth and differentiation) are profoundly influenced by cell shape and substrate adhesion/cell spreading (22,23).

### U0126 inhibits the proliferation of Mel-p cells

Cell shape perturbation, particularly that induced by cytoskeleton-disrupting drugs, alters the activity of specific signaling intermediates (24). Moreover, drug-initiated alterations in both the microfilament and microtubule networks also mobilize intracellular signaling elements and activate the ERK, JNK and p38 mitogen-activated protein kinases (MAPKs) (25,26). In a number of mammalian cell types, the Ras/MAPK cascade is the principal mitogenic signaling pathway and MAPK activation is essential for cell growth (27). Alesiani *et al* demonstrated that downregulation of the RAF/MEK/ERK pathway sensitizes melanoma cells to 5,7-dimethoxycoumarin treatment, accompanied by morphological changes including dendrite outgrowth (28).

**Figure 2.**
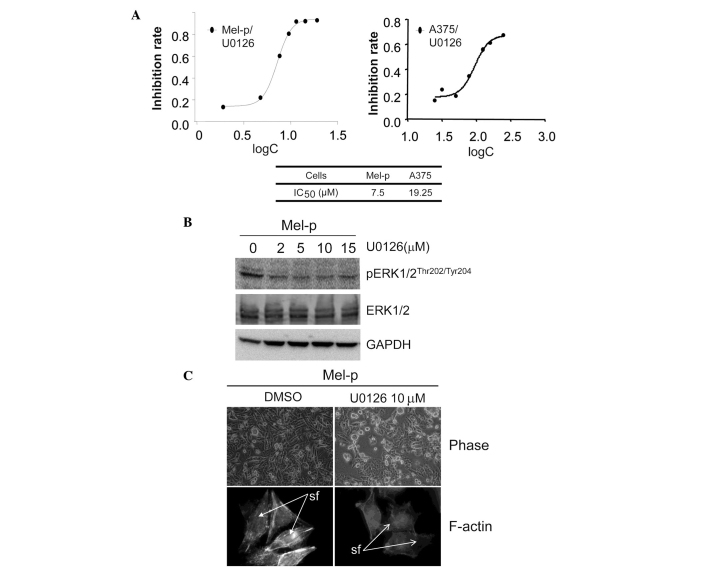
MEK inhibitor, U0126 suppresses cell proliferation, but has no effect on cell morphology. (A) Mel-p and A375 cells were treated with various concentrations of U0126 for 72 h. Cell viability was measured using the MTT assay, and the IC_50_ values of U0126 were determined. Results shown are representative of three independent experiments. (B) The levels of phospho-ERK1/2^Thr202/Tyr204^ and total ERK1/2 were assessed in Mel-p cells treated with various concentrations of U0126 for 24 h using western blot analysis. (C) Morphology and actin cytoskeleton arrangements in Mel-p cells induced by U0126.

To address whether there is an association between Src, MAPK and the actin cytoskeleton, the effect of ERK on cell proliferation and morphology was subsequently investigated. Treatment with the MEK inhibitor, U0126, resulted in a significant decrease in cell proliferation in Mel-p cells compared with vehicle control-treated cells. The IC_50_ value following a 72-h treatment was calculated (Fig. 2A). However, 20 *μ*M U0126 did not significantly decrease the growth of A375 cells. This result indicates that inhibition of primary melanoma cell growth by dasatinib may be associated with the activation of ERK. We demonstrated that ERK activity was significantly inhibited in Mel-p cell lines following treatment with the MEK inhibitor, U0126 (Fig. 2B). By contrast, U0126 exhibited almost no effect on cell morphology and the cytoskeleton. Notably, U0126 induced a level of cell rounding in Mel-p cells similar to that induced by dasatinib treatment (Fig. 2C). This suggests that part of the cytoskeletal remodeling induced by dasatinib is due to the inhibition of MEK activation.

### Dasatinib inhibits nuclear translocation of ERK signaling in Mel-p

Maat *et al* identified Src to be a crucial upstream tyrosine kinase for ERK1/2 activation in primary uveal melanoma (20), suggesting that Src-ERK1/2 signaling may be important for primary melanoma growth. A previous study confirmed the contribution of c-Src to cell shape-dependent ERK1/2 activation (29). It is also well known that growth stimulation by v-Src requires the activation of MEK/ERK signaling (30). Elements of the Ras/Raf/MAPK cascade associate with a microfilament-linked signaling ‘particle’, suggesting a cell structural basis for MAPK activation (31,32). v-Src-induced loss of stress fibers and morphological transformation have been demonstrated previously (33).

**Figure 3.**
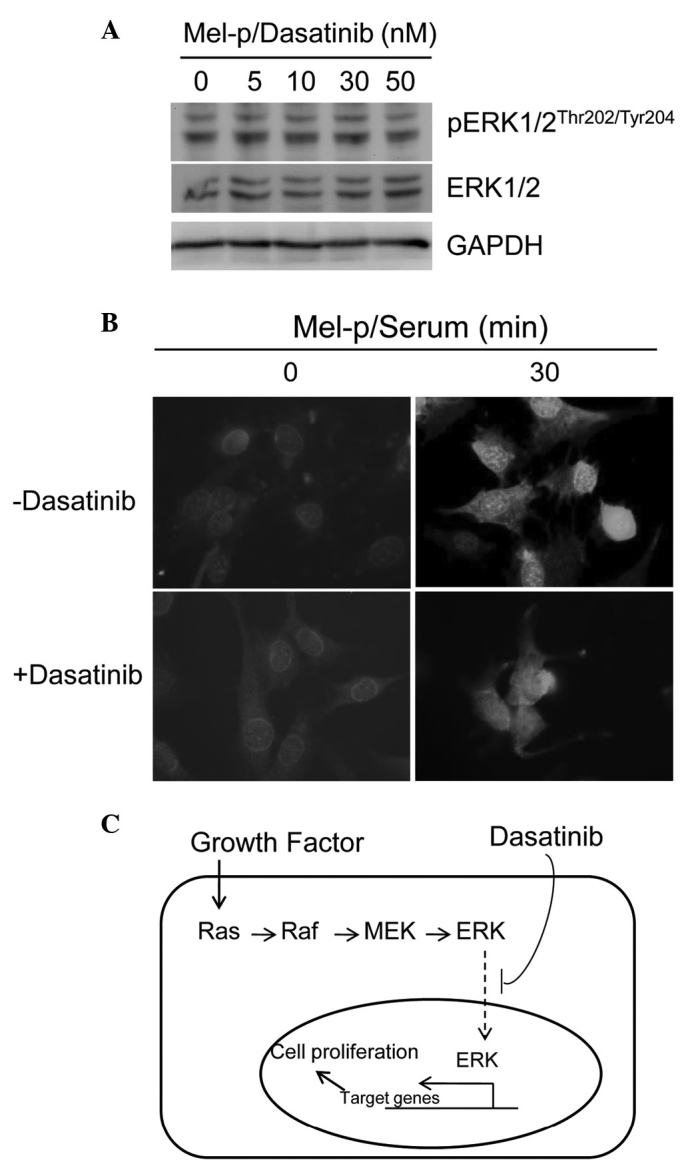
Dasatinib inhibits nuclear translocation of ERK1/2 in Mel-p. (A) The levels of phospho-ERK1/2^Thr202/Tyr204^ and total ERK1/2 were assessed in Mel-p cells treated with various concentrations of dasatinib for 24 h using western blot analysis. (B) Location of activated ERK1/2 by immunofluorescence microscopy. Mel-p cells were plated on glass coverslips in the presence or absence of dasatinib for 24 h, serum starved overnight and stimulated with 10% fetal bovine serum (FBS). At the indicated times, cells were fixed with 60% acetone/3.7% formaldehyde, stained with rabbit anti-phospho-ERK1/2^Thr202/Tyr204^ (green), and nuclei were stained with DAPI (blue). As shown in the upper panel, in control cells, nuclear localized p-ERK was detectable within 5 min and reached a maximum by 30 min, whereas the pERK1/2 in dasatinib-treated cells was mainly cytoplasmic at all times after serum addition. (C) Schematic depicting regulation of nucleocytoplasmic MAPK activity by dasatinib. The nuclear activity of ERK is required for mitogen-stimulated cell proliferation and dasatinib inhibits the nuclear translocation of ERK signaling. MAPK, mitogen-activated protein kinase.

Furthermore, the effects of dasatinib on Src-ERK signaling were evaluated in Mel-p cell lines in the present study. Dasatinib caused complete or near-complete inhibition of Src activity, as measured by phosphorylation at Y416 in western blot analysis following treatment overnight with concentrations ≥30 nM (Fig. 1D). However, no significant change in ERK phosphorylation was observed with dasatinib treatment (Fig. 3A), suggesting that ERK activation is not associated with Src inhibition.

Smith *et al* demonstrated that retinoic acid-induced differentiation of F9 cells results in uncoupling of MAPK activation and c-Fos expression (34). It was of interest to determine whether a similar regulation of the MAPK pathway occurs in Mel-p cells treated with dasatinib. To confirm that dasatinib-induced differentiation alters MAPK nucleo-cytoplasmic localization, activated pERK1/2 localization using indirect immunofluorescence microscopy was examined in the current study. In untreated cells, pERK1/2 was detectable in the nuclei within 5 min, reaching a maximum by 30 min and remaining visible 30–60 min after serum addition (Fig. 3B, upper panel). In dasatinib-treated cells, activated ERK1/2 was readily detected within 5 min (data not shown). However, the pattern of pERK1/2 cellular distribution was markedly different between untreated and treated cells. In dasatinib-treated cells, pERK1/2 was mainly distributed in the cytoplasm following serum addition (Fig. 3B, lower panel). Thus, nuclear translocation of activated pERK1/2 is impaired in dasatinib-treated cells, suggesting that dasatinib disrupts ERK1/2 signaling.

### Conclusions

Dasatinib has been demonstrated to be a differentiation-inducing compound in human multipotent mesenchymal stromal cells (35) and megakaryocytes (36). In the present study, we have demonstrated that dasatinib induces morphological (abored formation) differentiation in Mel-p cells. Several mechanisms have been proposed to explain the reduction in cell proliferation and impaired growth factor responsiveness that accompany differentiation. This study indicates that dasatinib induces differentiation and uncouples MAPK activation by suppressing the nuclear translocation of activated MAPK.

## Abbreviations:

SFK: Src tyrosine kinase family;
CML: chronic myelogenous leukemia;
ALL: acute lymphoblastic leukemia;
UM: uveal melanoma;
DMEM: Dulbecco’s modified Eagle’s medium;
FBS: fetal bovine serum;
BSA: bovine serum albumin;
MAPKs: mitogen-activated protein kinases
